# Editorial: Recent advances in spore forming pathogenic bacteria

**DOI:** 10.3389/fcimb.2026.1858596

**Published:** 2026-05-07

**Authors:** Pablo F. Pérez, Christina Nielsen-LeRoux

**Affiliations:** 1 Centro de Investigación y Desarrollo en Ciencia y Tecnología de los Alimentos, Centro Científico Tecnológico (CCT)-CONICET, La Plata, Argentina; 2 Cátedra de Microbiología. Facultad de Ciencias Exactas, Universidad Nacional de La Plata (UNLP), La Plata, Argentina; 3 Université Paris Saclay, Institut National de Recherche pour l'Agriculture, l'Alimentation et l´Environnement (INRAE), AgroParisTech, Micalis Institute, Jouy-en-Josas, France

**Keywords:** *Bacillus cereus*, *Clostridioides difficile*, pathogenesis, virulence factors, spore forming bacteria

Throughout the bacterial life cycle, numerous stress factors represent challenges that bacterial populations must overcome. Even during growth in nutrient-rich culture media in the laboratory, microbial populations face nutrient depletion, increased population density, and the accumulation of harmful metabolic byproducts. Without specific survival mechanisms, these factors would lead to the death of the majority of individuals within the population. Among the various strategies to address such stress, spore formation undoubtedly constitutes a successful evolutionary alternative.

The ability to produce spores brings about significant changes in how microorganisms interact with different environments, including humans and animals. In the case of pathogenic microorganisms, the capacity to sporulate facilitates dissemination and directly impacts the relationship with the host, consequently affecting virulence. It can be argued, that spore-forming bacteria and their hosts have co-evolved, developing strategies to optimize survival.

This Research Topic addresses different levels of the interaction between representative spore-forming pathogenic bacteria and their hosts. A review of the articles in this Research Topic indicate that the environment selects strains with particular capabilities, however of note *in vivo* outcomes may not always be predictable by *in vitro* assays. Furthermore, the analysis of the genetic repertoire, combined with various experimental tools, can help pinpoint characteristics relevant to pathogenesis.

Considering the versatility of pathogens such as *Bacillus cereus*, which is capable of producing harmful effects in both intestinal and extraintestinal environments, the work by Coburn et al. reveals that the genetic makeup of *B. cereus* strains varies according to their origin. For instance, the genes *capK, cytK, hblA, hblC*, and the master virulence regulator (PlcR) were found in more than 93% of ocular strains, while their frequency was lower in gastrointestinal isolates. Other genes, such as *nheA, nheB, inhA1, inhA2, and sodA1*, were common across strains of different origins. Regarding *in vitro* biological activity, digestive strains exhibited higher hemolytic activity than ophthalmic ones. However, when tested in an *in vivo* model of endophthalmitis, no differences were observed based on the origin of the strains. Moreover, intraocular concentrations were similar when comparing a selected ocular strain with gastrointestinal isolates. Interestingly, all ocular strains were positive for the *capK* gene—related to capsule synthesis—while only 11.1% of digestive strains carried this gene.

Beyond the fact that certain bacterial species can produce a wide array of virulence factors, in some cases, microorganisms are capable of refining their repertoire of secreted factors, giving rise to isoforms that add new dimensions to host interaction. The work of Kranzler et al. shows that emetic strains *of B. cereus* can produce different variants of cereulide (isocereulides). Through a biotechnological approach to produce cereulide in large quantities, they demonstrated that the reference emetic strain F4810/72 can produce 13 isoforms in addition to the canonical cereulide. Furthermore, they showed that the variants produced in the highest proportions are not necessarily responsible for the greatest biological activity.

The works of Minnaard et al. and Barbero et al. add another dimension to this landscape. Both studies consider not only microbial aspects but also the host responses. They achieve this through investigations into the influence of platelets on the *in vitro* phagocytosis of *Clostridioides difficile* by human peripheral blood-derived macrophages, and through an invertebrate model (*Galleria mellonella*) of *Bacillus cereus* infection. Both studies illustrate the “two sides” of the immune response—described by Barbero et al. as a “double-edged sword”—which can serve to control an infection but may also trigger damage through an exacerbated inflammatory response. This is a likely scenario when microorganisms gain access to unusual environments due to their colonization capabilities.

The study by Barbero et al. introduces a new protagonist to the story: platelets, which are abundant in tissues damaged by inflammation (a hallmark of *C. difficile* infection). The authors demonstrate that the presence of platelets promotes the internalization *of C. difficile* by macrophages under non-opsonic conditions through a process of macropinocytosis. This is particularly relevant, as the internalization pathway often determines the subsequent immune response. Within this context of host response, Minnaard et al. utilize the larval insect model (*G. mellonella)* to dissect the innate immune response against two foodborne *B. cereus* strains with different activities in *in vitro* models. Their work suggest that genes and regulatory mechanisms different from canonical *B. cereus* virulence factors could play a significant role in host interaction. Indeed, two strains belonging to different genetic groups resulted in two markedly different phenotypes in the insect: an “aggressive” phenotype (associated with strain B10502) and a “persistent” phenotype (associated with strain T1). These findings confirm and enhance the differences observed for these strains in *in vitro models.*

The contributions to this Research Topic highlight two key aspects of the pathogen-host relationship: adhesion/invasion phenomena and the production of extracellular factors. The findings of each of these studies provide a foundation for future work aimed at understanding the mechanisms mediating pathogenesis, as well as cornerstones for the control of sporeformer pathogens.

